# SONOPS Multicentre Cross-Sectional Study: Study of the Nitrous Oxide Perception and Use in French Dental Students

**DOI:** 10.1016/j.identj.2025.02.007

**Published:** 2025-02-19

**Authors:** Mélanie Duval, Maud Rodney, Morgane Rousselet, Choosie Jaquin, Elsa Garot, Thomas Marquillier, Ariane Camoin, Marion Strub, Mathieu Marty, Edouard-Jules Laforgue, Caroline Victorri-Vigneau, Tony Prud'homme

**Affiliations:** a Nantes Université, CHU Nantes, Centre d'Evaluation et d'Information sur la Pharmacodépendance-Addictovigilance (CEIP-A), Service de Pharmacologie Clinique, Nantes, France; b Nantes Université, CHU Nantes, Odontologie pédiatrique, Nantes, France; c Nantes Université, Univ Tours, CHU Nantes, CHU Tours, INSERM, MethodS in Patients-Centered Outcomes and HEalth Research, SPHERE, Nantes, France; d Université de Bordeaux, CHU Bordeaux, Odontologie pédiatrique, Bordeaux, France; e Université de Lille, CHU Lille, Odontologie pédiatrique, Lille, France; f Université Sorbonne Paris Nord, Laboratoire Éducations et Promotion de la Santé, Villeaneuse, France; g Aix Marseille Univ, CHU Marseille, CNRS, EFS, ADES, Marseille, France; h Université de Strasbourg, CHU Strasbourg, Odontologie pédiatrique, Strasbourg, France; i Université de Toulouse, CHU Toulouse, Odontologie pédiatrique, Toulouse, France; j Nantes Université, CHU Nantes, INSERM, Regenerative Medicine and Skeleton, RMeS, Nantes, France

**Keywords:** Nitrous oxide, Prevalence of use, Dental students, Recreational use

## Abstract

**Introduction and aims:**

EMONO (equimolar oxygen-nitrous oxide mixture) is widely used in dentistry to achieve sedation for dental care. In addition, pure nonmedical nitrous oxide (N2O) has become a very popular psychoactive substance among health students. Thus, for dental students, the perception of a same substance, N2O, which can either be used as medicine in the form of EMONO in their daily practice, or consumed illegally for recreational purposes in the form of pure nonmedical N2O, is of concern. This study aims to estimate the prevalence of N2O (EMONO and pure nonmedical N2O) use among French dental students.

**Methods:**

A cross-sectional multicentre study was carried out in six French dental schools. A self-administered questionnaire was offered to 2nd to 6th-year odontology students about their position with regard to N2O and its potential use.

**Results:**

The prevalence of N2O use among the 1124 responding students was 50.4%, with heterogeneity according to dental school. 84% of the students who used N2O did so at least once for recreational purpose, while 16% used it only in the context of care and/or training. Students with recreational N2O use were more likely to use other substances than students with care/training use. Nearly three-quarters of the students sought and experienced euphoria and laughter.

**Conclusion:**

Nearly half the students in our study reported having used N2O recreationally, most of them regularly, a much higher prevalence than among nonhealthcare students.

**Clinical relevance:**

The issue of the correct use of EMONO is particularly important at a time of increasing detour from medical to recreational use. As future healthcare professionals with easy access to this substance, dental students should be well versed in the use and abuse of nitrous oxide in dentistry.

## Introduction

### Nitrous oxide and dentistry

Nitrous oxide (N2O) mixed with oxygen (equimolar oxygen-nitrous oxide mixture: EMONO) can be used in France to achieve sedation for dental care. It obtained approval in 2001 for its use in dental care of children and patients with anxiety or disability in hospitals exclusively. EMONO has analgesic and anxiolytic properties, and then constitutes a reference to help the realization of dental care in uncooperative paediatric patients.^1^, ^2^, ^3^, ^4^ Its pharmacokinetic properties allow a rapid effect and quick return to the initial state of consciousness after discontinuation.^5^, ^6^, ^7^, ^8^, ^9^ It also facilitates patient treatment and prevents the development of possible long-term anxiety related to dental care. In France, since 2009, dentists are authorized to use it in their dental offices with specific training (EMONO attestation) approved by the national authorities.^10^ General teachings on EMONO and pure N2O are also provided by all dental schools during their university curriculum, at different times depending on the dental school.

### Nitrous oxide and medical students

In recent years, pure nonmedical N2O has become one of the most widely used psychoactive substances among young people, particularly health students. This dramatic increase in consumption is associated with harmful consequences such as neurologic, cardiovascular, psychiatric and addictive risks.^11^^,^^12^ Substance Use Disorders (SUD) are a topic of concern in this specific population.^13^ Cohen and colleagues performed a descriptive, cross-sectional and observational study about the prevalence of N2O use and N2O use disorders among 981 French medical students: out of the 981 medical students (29% of the total medical students) who completed the questionnaire, 80% had used N2O. The incidence of SUD was 19% for mild cases, 4% for moderate cases, and 1% for severe cases.^14^ Other studies on the subject exist.^15^^,^^16^

### Dental students, pure nonmedical N2O and EMONO, which use?

Thus, and particularly for dental students, the perception of a same substance, N2O, that can be either used as a medicine in the form of EMONO in their daily practice, easily available, and either used illegally for recreational purposes in the form of pure nonmedical N2O, is of concern. It is therefore important to understand how dental students use nitrous oxide. Do they use nitrous oxide solely in a medical or training context? Or do some of them also use nitrous oxide for recreational purposes? If so, what effects are they looking for? The side effects of nitrous oxide, such as altered sensory perceptions or euphoria, as well as the rapid onset of these effects and return to a normal state of consciousness, are some of the reasons why students may use it for recreational purposes.

The main objective of this study was to estimate the prevalence of nitrous oxide use – EMONO and pure nonmedical N20 – among French dental students. The secondary objectives were to describe the students who used nitrous oxide, to describe the nitrous oxide patterns, to assess the sought effects of nitrous oxide and the effects actually experienced, and finally to compare these effects with those experienced when using other psychoactive substances.

## Methods

### Study design and population

This cross-sectional, descriptive, multicentre study was carried out at six French dental schools (Bordeaux, Lille, Marseille, Nantes, Strasbourg and Toulouse). These dental schools were chosen for the study because they are among the largest of the 15 French dental schools with regard to the number of students trained, and were used to working with the Nantes dental school. Based on the product characteristics of EMONO as described in the marketing authorization and on literature data, a self-administered questionnaire was drawn up by a multidisciplinary team of dentists, pharmacologists, and research staff. The questionnaire was distributed to dental students in May-June 2019 for the Nantes dental school and over the 2021 to 2022 academic year for the other five dental schools. Students included were all students over 18 years of age in these six dental schools, from the 2nd to the 6th-year of study. The 2nd year of study corresponds to the beginning of the dentist's training, the 1st year of study being common in France to dentists, doctors, pharmacists, and midwives.

In accordance with current French law, oral information was provided to participants. The study received a favourable opinion from an ethics committee (Nantes Health Ethics Group – GNEDS). The study was registered on clinicaltrials.gov under the number NCT0405494.

### Data collection

The anonymous questionnaire was distributed in paper format to students. It was structured in two parts: (1) a first part collecting demographic data and information on students’ position with regard to N2O (training, interest in professional use, opinion on the legislative framework, previous use), (2) a second part, accessible only to students who had previously consumed N2O (EMONO and/or pure nonmedical N2O), collecting data on the way N2O was used (context, number, and frequency of uses), on the effects sought and experienced when using N2O, and on the similarity of the effects experienced with N2O to those experienced with other psychoactive substances.

### Outcomes

*a. Primary outcome*

The primary outcome was the prevalence of N20 use, i.e., the proportion of dental students who had used N2O – in the form of EMONO and/or pure nonmedical N2O – at least once in their lifetime.

*b. Secondary outcomes*

The first secondary outcome was the proportion of characteristics of dental students using nitrous oxide: gender, completion of EMONO training, interest in professional use of EMONO, opinion on the legislative framework regarding EMONO, and history of use of other psychoactive substances.

The second secondary outcome was the proportion of N2O use patterns: context of use, number of times used, and frequency of use.

The third secondary outcomes were the number of effects sought/experienced by the students, the type of effects sought/experienced, and the perception of the effects experienced (pleasant or unpleasant). The effects were separated into two types: festive effects (euphoria, laughter, visual distortion, auditory distortion, tactile distortion, voice modification) and therapeutic effects (analgesia, anxiolysis). The evaluation of the experienced effects also included the description of side effects (nausea, amnesia, sleepiness, headaches, vertigo, and tinnitus).

The fourth secondary endpoint was, for each other psychoactive substance consumed, the proportion of students reporting having experienced effects similar to those of N2O when experimenting with the psychoactive substance studied. The psychoactive substances studied were presented in a list form, corresponding to the substances most commonly consumed by students.^17^

### Statistical analysis

The prevalence of N2O use was estimated with its 95% confidence interval (CI). The population of students who had used N2O exclusively in a care or training context and that of students who had used it at least once in a recreative context were separated in order to highlight their different consumption habits. In the following sections, the former will be referred to as ‘care/training users’ and the latter as ‘recreational users’. Their responses were compared using t-tests for quantitative variables, Chi2 tests, or Fisher's exact tests for qualitative variables. A two-tailed significance level of 5% was used. All the analyses were carried out using R version 4.02.

## Results

### Study population

Of the 1124 students who completed the questionnaire, 60% (*n* = 679) were women. Although 64% of students explained they were interested in the use of EMONO in their professional practice, only 34% declared having received training in the use of EMONO. Moreover, 62% considered that current French legislation was sufficient to limit the risk of abuse by healthcare professionals. Responses to these three questions varied according to respondents’ dental school (*P* < .001).

### Prevalence of nitrous oxide use

The prevalence of N2O use during their lifetime was 50.4% (47.5%; 53.4%) (*n* = 567), with heterogeneity according to dental school (from 29% to 81%, *P* < .001). Among these students, 84% (*n* = 475) had used N2O at least once for recreational purpose while 16% (*n* = 91) said they had used N2O only in the context of care and/or training (in the form of EMONO) but never recreationally.

### Characteristics of students using nitrous oxide

The characteristics of students according to the context of their N2O consumption are detailed in Table 1. The proportion of men was significantly higher among recreational users than care/training users only and nonusers (47% vs 37 and 33%, *P* < .001). Recreational users declared also more significantly that the current French legislative framework is insufficient to limit abuse by healthcare professionals (43% vs 35% and 34%).

**Table 1:** Characteristics of the 1124 dental students according to the context of their nitrous oxide consumption.

	Care/training users* (*n* = 91)	Recreational users† (*n* = 475)	Nonusers (*n* = 557)	*P* value
Gender:				<.001
Men (*n*, %)	34 (37.4%)	224 (47.2%)	186 (33.4%)	
Women (*n*, %)	57 (62.6%)	251 (52.8%)	371 (66.6%)	
EMONO training (*n*, %)	39 (42.9%)	163 (34.3%)	180 (32.3%)	.142
Interest in professional use (*n*, %)	63 (69.2%)	298 (63.0%)	356 (64.6%)	.513
Opinion on legislative framework: Insufficient (*n*, %)	29 (34.5%)	194 (43.2%)	175 (34.2%)	.013

Missing data: <5% for context of nitrous oxide consumption, <5% for interest in professional use, <10% for opinion on legislative framework.

⁎ N2O users only for care or training (EMONO).

† N2O users with at least one recreational use.

Students with recreational N2O use were more likely to use other substances than students with care/training use. They reported using significantly more frequently alcohol (89% vs 78%), tobacco (68% vs 46%), cannabis (64% vs 42%), cocaine (17% vs 4%), ecstasy/MDMA (23% vs 9%) and poppers (63% vs 39%). These substances, along with tramadol and opioid analgesic were the most widely consumed by N2O-using students. Details of all substance uses are given in Table 2.

**Table 2:** Psychoactive substances use of the 567 dental nitrous oxide-using students.

	Care/training users (*n* = 91)	Recreational users (*n* = 475)	*P* value
Tobacco (*n*, %)	42 (46.1%)	321 (67.6%)	<.001
Alcohol (*n*, %)	71 (78.0%)	422 (88.8%)	.005
Cannabis (*n*, %)	38 (41.8%)	303 (63.8%)	<.001
Stimulants (*n*, %)			
Cocaïne	4 (4.4%)	82 (17.3%)	.002
Crack/freebase	0 (0.0%)	5 (1.0%)^1^	1.000
Amphetamines	3 (3.3%)	37 (7.8%)	.126
Cristal meth	0 (0.0%)	7 (1.5%)	.605
Ecstasy/MDMA	8 (8.8%)	111 (23.4%)	.002
DMT	1 (1.1%)	6 (1.3%)	1.000
Opioids (*n*, %)			
Heroin	1 (1.1%)	2 (0.4%)	.410
Tramadol	16 (17.6%)	109 (22.9%)	.258
Opioid analgesic tablets	19 (20.9%)	93 (19.6%)	.776
Volatile substances (*n*, %)			
Poppers	35 (38.5%)	298 (62.7%)	<.001
GHB/GBL	1 (1.1%)	6 (1.3%)	1.000
Hallucinogenic mushrooms (*n*, %)	10 (11.0%)	52 (10.9%)	.991
Ketamine (*n*, %)	2 (2.2%)	30 (6.3%)	.119
LSD (*n*, %)	1 (1.1%)	24 (5.0%)	.157
NPS (*n*, %)	0 (0.0%)	6 (1.3%)	.596
Benzodiazepines (*n*, %)	9 (9.9%)	36 (7.6%)	.455
Pregabalin (*n*, %)	0 (0.0%)	2 (0.4%)	1.000
Ayahuasca (*n*, %)	1 (1.1%)	3 (0.6%)	.505

Missing data: <5% for crack/freebase use.

### Patterns of nitrous oxide use

The patterns of N2O use are described in Table 3. Of the students who had used N2O for recreational use 18% had also benefited from N2O as part of their care (*n* = 87) and 10% as part of their training (*n* = 46). Nearly a quarter of recreational users students have used it only once in their lives compared with 85% of care/training users (*P* < .001). 38% of recreational users have used N2O more than 10 times. In terms of frequency of use, the vast majority (84%) of recreational users reported using N2O less than once a month, while 4% used it weekly or daily. Care/training users were much more occasional users (92%), in accordance with the context in which they used the product (*P* < .001).

*a. Type of effects sought and experienced*

**Table 3:** Patterns of nitrous oxide use of the 567 dental nitrous oxide-using students.

	Care/training users *n* = 91)	Recreational users (*n* = 475)	*P* value
Use context (*n*, %)			NA
Care	64 (70.3%)	87 (18.3%)	
Training	31 (34.1%)	46 (9.7%)	
Type of care (*n*, %)			NA
Dental care	12 (13.2%)	29 (6.1%)	
Medical care	55 (60.4%)	68 (14.3%)	
Number of uses (*n*, %)			<.001
1 time	77 (84.6%)	111 (23.4%)	
Between 2 and 10 times	11 (12.1%)	184 (38.7%)	
More than 10 times	3 (3.3%)	180 (37.9%)	
Frequency of use (*n*, %)			<.001
Occasional use	84 (92.3%)	201 (42.3%)	
<1 time/mo	5 (5.5%)	199 (41.9%)	
≥1 time/mo	2 (2.2%)	58 (12.2%)	
≥1 time/wk	0 (0.0%)	15 (3.2%)	
Everyday	0 (0.0%)	2 (0.4%)	

The vast majority of recreational users (88%, *n* = 416) were looking for at least one effect (87% at least one festive effect; 15% at least one therapeutic effect). In contrast, less than half of care/training users (46%, *n* = 42) were looking for at least one effect (festive effect: 15%; therapeutic effect: 39%).

The two main festive effects sought when using N2O were euphoria and laughter (79% and 59%, respectively of recreational users). These two effects were also the most experienced by these students (75% and 63%, respectively). The therapeutic effect most sought and experienced by all students was analgesia (experienced by 43% of care/training users and 18% of recreational users). For both care/training and recreational users, the proportion of students who experienced each type of festive effect was greater than the proportion of students who sought the same type of effect, except for euphoria, with a difference in proportions of up to 30% (Figure). The same phenomenon was observed for therapeutic effects, albeit in smaller proportions.

**Fig:**
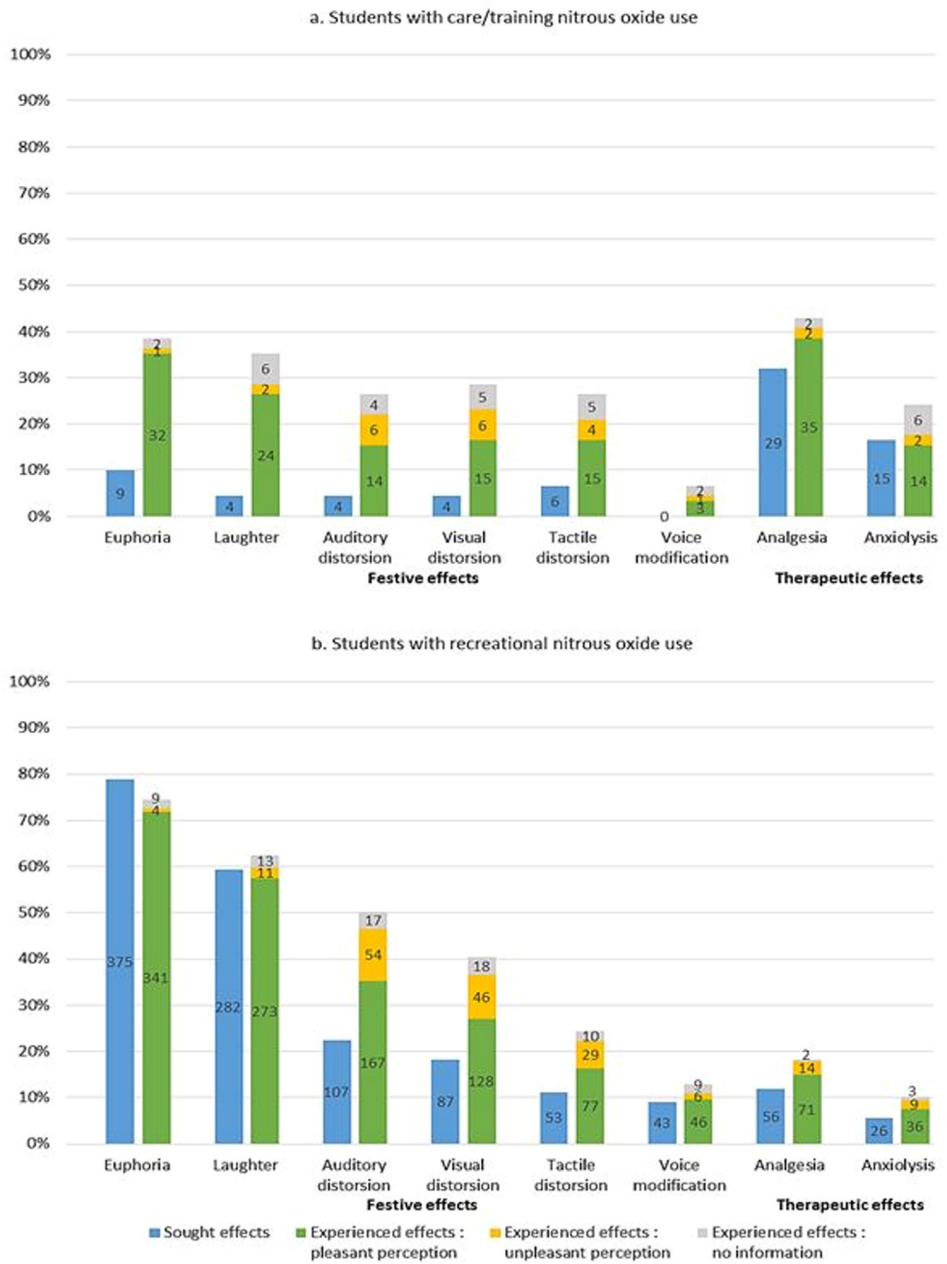
Comparison of sought and experienced effects by the 567 dental nitrous oxide-using students.

The side effects most often experienced by students were vertigo: 22% of care/training users and 23% of recreational users. The proportion of side effects experienced was similar for both student populations, with the exception of sleepiness (25% of care/training users but only 8% of recreational users). Other side effects were experienced in smaller proportions (headaches: 13% and 16% for care/training users and recreational users, respectively; tinnitus: 10% and 13%, respectively; amnesia: 9% and 5%, respectively; nausea: 4% and 5%, respectively).

*b. Exploration of the perception of therapeutic and festive effects*

Euphoria and laughter, although not initially sought-after, were perceived as pleasant by almost all students (Figure). Other festive effects were perceived as unpleasant by around a quarter of students. Although more care/training users perceived analgesia and anxiolysis positively, these two therapeutic effects were perceived favourably by at least 80% of students.

*c. Similarities between effects of nitrous oxide and effects of other psychoactive substances*

The psychoactive substance that is declared to provide the most N2O-like effects was poppers, cited by 47% of the 333 students who had experimented with both poppers and N2O (Table 4). The other psychoactive substances were all cited by less than 20% of students who experimented with them, with the exception of crystal meth and heroin, which were very rarely experimented with by students (*n* = 2/7 and *n* = 1/3, respectively). The effects of alcohol and ketamine were described as similar to those of N2O by 19% of students (*n* = 96/494 and *n* = 6/32, respectively).

**Table 4:** Comparison of the effects of nitrous oxide and other psychoactive substances used by the 567 dental nitrous oxide-using students.

Other psychoactive substances used by students (*n* = 527)	Students declaring an N20 like effect with this substance
Tobacco *n* = 363/527 (68.9%)	9/363 (2.5%)
Alcohol *n* = 494/527 (93.7%)	96/494 (19.4%)
Cannabis *n* = 341/527 (64.7%)	50/341 (14.7%)
Stimulants	
Cocaine *n* = 86/527 (16.3%)	1/86 (1.2%)
Crack/freebase *n* = 5/527 (0.9%)	0/5 (0.0%)
Amphetamines *n* = 40/527 (7.6%)	2/40 (5.0%)
Cristal meth *n* = 7/527 (1.3%)	2/7 (28.6%)
Ecstasy/MDMA *n* = 119/527 (22.6%)	17/119 (14.3%)
DMT *n* = 7/527 (1.3%)	1/7 (14.3%)
Opioids	
Heroin *n* = 3/527 (0.6%)	1/3 (33.3%)
Tramadol *n* = 125/527 (23.7%)	16/125 (12.8%)
Opioid analgesic tablets *n* = 112/527 (21.2%)	9/112 (8.0%)
Volatile substances	
Poppers *n* = 333/527 (63.2%)	155/333 (46.5%)
GHB/GBL *n* = 7/527 (1.3%)	0/7 (0.0%)
Hallucinogenic mushrooms *n* = 62/527 (11.8%)	8/62 (12.9%)
Ketamine *n* = 32/527 (6.1%)	6/32 (18.8%)
LSD *n* = 25/527 (4.7%)	2/25 (8.0%)
NPS *n* = 6/527 (1.1%)	0/6 (0.0%)
Benzodiazepines *n* = 45/527 (8.5%)	6/45 (13.3%)
Pregabalin *n* = 2/527 (0.4%)	0/2 (0.0%)
Ayahuasca *n* = 4/527 (0.8%)	0/4 (0.0%)

Missing data: <5% for cocaine.

## Discussion

### Prevalence of nitrous oxide use

The prevalence of N2O use during student's lifetime, in the form of EMONO and/or pure nonmedical N2O, was 50.4% in our study, with 42.3% involved in recreational use. The great heterogeneity in the prevalence of N2O consumption by dental students between the different dental schools could be explained by various factors: (1) regional disparities in pure nonmedical N2O consumption (an unverifiable hypothesis, since in France precise regional data on pure N2O consumption by the general population are not currently available), (2) heterogeneity in the prevention actions carried out by dental schools and student associations on the risks associated with the consumption of psychoactive substances, including nitrous oxide, and (3) general teachings on EMONO provided to students varying according to dental schools.

In comparison with other recent French studies, this is lower than that found among medical students in Paris (80%) or health students in Montpellier (76.6%),^14^^,^^15^ but much higher than among French students as a whole (26%). The substance's accessibility, combined with its innocuous reputation, leads a large number of students to use it recreative purpose. Being a health student seems to favour this type of consumption in France. If we take international examples, on New Zealanders 1st-year students (12%), among Dutch adolescent (15.6%) or in British general student (38,8%), the percentages of N2O lifetime use are lower.^16^^,^^18^^,^^19^ However, in the absence of studies on international healthcare students, apart from older studies, it is difficult to draw conclusions.^20^ Furthermore, in our study, the proportion of men was higher among students with recreational N2O use comparing with nonrecreational users. This is consistent with the Paris study on health students.^14^

### Nitrous oxide use patterns

38% of recreational users have used N2O more than 10 times and 4% used it weekly or daily. The consumption of some students is therefore particularly problematic, as shown in the study by Cohen et al^14^ in which 1% of students’ presented a severe use disorder. These students don't seem to be aware of the possible dangers of regular consumption. Furthermore, in our study, the proportion of unpleasant perceptions was high, even among recreational users. Thus, in this sample, recreative uses were likely to be repeated whereas negative consequences are experienced. Prolongated and repeated uses despite consequences are items of the DSM-5-TR^21^ definition of a SUD. It therefore seems necessary to keep emphasizing the risks of nitrous oxide consumption to the student population.

According to a previous report in French population,^17^ cannabis and poppers are the two mains illicit psychoactive substances consumed. Our results are in line with this report since around 60% of all N2O users in our sample consume these two substances. This rate is higher than the prevalence reported in a 2017 French survey among people from 18 to 25 (at least one use of popper: 14%). Tramadol, opioid analgesic tablets, and Ecstasy/MDMA were also consumed by a fifth of N2O users students. This suggests that dental students who use N2O consume more psychoactive substances than the general population. Multiple substance consumption is a known factor associated with SUD.

### Effects sought and experienced when using nitrous oxide

Euphoria and laughter are the main effects of N2O. Nitrous oxide used for nonmedical purposes has the same effects as EMONO. In a French survey published by the Nantes Addictovigilance Centre, which is responsible of surveillance of abuse, dependence, and consequences of N2O use in France, the evolution of the effects sought when using N2O was highlighted.^13^ We have recently observed a transition from festive use with a search for euphoria to consumption for self-therapeutic purposes with a search for amnesia or well-being; moreover, consumption in violent contexts, such as prostitution, is increasing. These evolutions are all the more worrying for future dentists, as they will potentially have gas cylinders available in their practices. There have already been reports of abuse and misuse by healthcare professionals, particularly dentists.^22^ The non-negligeable presence of pleasantly perceived nontherapeutic effects during training or practice in our results shed lights on these risks. In this context, a dedicated and mandatory training for professional use of N2O is essential to limit misuse for practitioners. During this training, the properties of the gas are developed as well as the risks, in particular the addictive risk. The EMONO risk management plan includes monitoring the quantities delivered to each professional. In addition, the possibility for dentists to use the gas in the course of their work is subject to the completion of training validated by the Dentist National Council.

### Comparison with the effects of other substances

For almost half of the subjects who consumed N2O and poppers, a similarity of effect was declared. As their frequent use in a student population, N2O and poppers shared some characteristics, notably pharmacologic ones. They are both consumed by inhalation, have short half-lives and produce transient euphoria that is the main sought and experienced effect for recreative consumers.^13^^,^^23^ These two substances are also at risk for complications.^13^^,^^24^^,^^25^ Pharmacology of N2O enhanced μ-opioid receptor and also GABA and NMDA to produce the analgesia and anxiolysis sought in therapeutic.^13^ The effect-like of other μ-opioid receptor agonists are contrasted, from 33% for heroïne (but for only 1/3 consumers) to 8% to 13% for opioïd and tramadol consumers. Similarity is declared for 10% to 20% of co-consumers for Ketamine, an NMDA receptor antagonist dissociative, alcohol and benzodiazepines, two GABA receptors modulators. Discrepancy of effect-like substances between recreative and therapeutic effects can reflect the singularity of N2O mechanism of action but also may be explained by the proportion of subjects with at least one recreative use (>80%) among all N2O consumers.

### Professional use of EMONO

Few French dentists use EMONO in their practice, and many do not treat children at all,^26^ so patients needing specific treatment under sedation experience long delays and even breakdowns in care, and struggle to find practitioners. In our study, 34% of students say they have received training in the use of EMONO. Given that the students were interviewed during their studies, and that training differs from one centre to another, it's not illogical to find that few students are already trained. What is encouraging, however, is that 64% of students said they were interested in the use of EMONO in their professional practice. We can therefore hope that, even in the event of a training shortfall, future practitioners will continue their training once they have completed their studies.

A sizeable proportion of students either didn't give an opinion on the current legislative framework for nitrous oxide or couldn't decide, probably because they are unaware of the institutional rules surrounding the medical use of EMONO, and possibly of the lack of supervision of pure nitrous oxide available over the counter. Despite this, 62% felt that current French legislation was sufficient to limit the risk of abuse by healthcare professionals. It's worth noting that this information needs to be passed on even more widely, especially as there are differences between study city in this aspect.

### Strengths and limitations

Our study has the advantage of interviewing a very broad panel of dental students (>1000 students from 6 different dental school). Anonymity enabled us to obtain honest responses, with some students describing significant consumption.

It is to be hoped, therefore, that the declarative and subjective aspect of the study is as unbiased as possible in drawing conclusions about N2O consumption. For future studies on N2O consumption in the population of health students, it could be interesting to carry out subgroup analyses based on years of study. This would make possible to see if consumption trends decrease based on knowledge about the addictive potential of the substance.

## Conclusions

The issue of the correct use of EMONO is particularly important at a time of increasing detour from medical to recreational use. As future healthcare professionals with easy access to this substance, dental students are particularly concerned by this issue. Thus, nearly half the students in our study reported having used N2O recreationally, most of them regularly, a much higher prevalence than among nonhealthcare students.

## Conflict of interest

The authors declare that they have no competing interests.
